# Immunomodulatory and Anti-IBDV Activities of the Polysaccharide AEX from *Coccomyxa gloeobotrydiformis*

**DOI:** 10.3390/md15020036

**Published:** 2017-02-10

**Authors:** Qiang Guo, Qiang Shao, Wenping Xu, Lei Rui, Ryo Sumi, Fumio Eguchi, Zandong Li

**Affiliations:** 1 State Key Laboratory for Agrobiotechnology, College of Biological Sciences, China Agricultural University, Beijing 100193, China; fzgq249@163.com (Q.G.); shaoqiang19880316@126.com (Q.S.); xwp120@126.com (W.X.); ruilei@cau.edu.cn (L.R.); 2 Nikken Sohonsha Corporation, Gifu 501-6255, Japan; ryosumi_nikken@yahoo.co.jp; 3 Faculty of Regional Environment Science, Tokyo University of Agriculture, Tokyo 156-8502, Japan; f1eguchi@nodai.ac.jp

**Keywords:** *Coccomyxa*, polysaccharide, IBDV, AEX, cytokine

## Abstract

A number of polysaccharides have been reported to show immunomodulatory and antiviral activities against various animal viruses. AEX is a polysaccharide extracted from the green algae, *Coccomyxa gloeobotrydiformis*. The aim of this study was to examine the function of AEX in regulating the immune response in chickens and its capacity to inhibit the infectious bursal disease virus (IBDV), to gain an understanding of its immunomodulatory and antiviral ability. Here, preliminary immunological tests in vitro showed that the polysaccharide AEX can activate the chicken peripheral blood molecular cells’ (PBMCs) response by inducing the production of cytokines and NO, promote extracellular antigen presentation but negatively regulate intracellular antigen presentation in chicken splenic lymphocytes, and promote the proliferation of splenic lymphocytes and DT40 cells. An antiviral analysis showed that AEX repressed IBDV replication by the deactivation of viral particles or by interfering with adsorption in vitro and reduced the IBDV viral titer in the chicken bursa of Fabricius. Finally, in this study, when AEX was used as an adjuvant for the IBDV vaccine, specific anti-IBDV antibody (IgY, IgM, and IgA) titers were significantly decreased. These results indicate that the polysaccharide AEX may be a potential alternative approach for anti-IBDV therapy and an immunomodulator for the poultry industry. However, more experimentation is needed to find suitable conditions for it to be used as an adjuvant for the IBDV vaccine.

## 1. Introduction

Polysaccharides present an enormous variety of structures in organisms and are an under-exploited novel source of natural compounds for drug discovery. Plant and marine microalgae-derived polysaccharides have been shown to have a variety of bioactivities, such as immune-modulatory, anti-viral, anti-tumor, anticoagulant, and antioxidant properties [1]. The polysaccharide from Astragalus radix can increase the production of TNF, GM-CSF, and NO in mice [2]. The sulfated polysaccharide, p-KG103, which is purified from the marine microalgae, *Gyrodinium impudium*, can activate NO production in a JNK-dependent manner to stimulate the production of cytokines, such as interleukin-1 (IL-1), IL-6, and TNF-α, in macrophages, thereby preventing tumor cell growth both in vitro and in vivo [3,4]. Polysaccharides are also crucial regulatory factors for the adaptive immune response, especially humoral immunity. Jingjing Yang et al. reported that a water-soluble polysaccharide (WSPA), which is isolated from the stem of *Physalis alkelengi* L., significantly enhanced specific antibody IgG titers in mice [5]. The polysaccharides derived from Taishan *Pinus massoniana* pollen (TPPPS) also improved the effects of different vaccines when used as an immunoadjuvant [6,7,8]. In addition, several types of polysaccharides (such as carrageenan, alginate, fucan, laminarin, ulvan, dextran sulfate, heparin, and fucoidan (fuc)) possess a broad-spectrum of antiviral activity in vitro against dengue virus, herpes simplex virus (HSV), human immunodeficiency virus (HIV), and influenza virus by targeting several steps of the viral cycle [9,10,11,12,13,14,15,16].

Infectious bursal disease virus (IBDV) is the etiological agent of infectious bursal disease (IBD), which is an acute, highly contagious disease in young chickens [17] that contributes to a huge economic loss in the public poultry industry. IBDV is a non-enveloped, double-stranded RNA (dsRNA) virus belonging to the *Birnaviridae* family [18,19,20]. IBDV infection causes a massive destruction of B cells in lymphoid organs, resulting in lymphopenia (immunosuppression) [21]. This leads to an increased susceptibility to secondary infection [22]. 

*Coccomyxa gloeobotrydiformis* is a species of green algae that was first isolated in 1969 by Reisigl [23]. It belongs to the family of Coccomyxaceae and distributes in Iceland, Himalayas, Japan, New Zealand and Antarctica [24]. Previous studies have shown that *Coccomyxa gloeobotrydiformis* has a variety of bioactivities in a rat model, such as neuroprotective effects in ischemic stroke [25], learning and memory improvement effects in intrinsic aging rats [26], inhibitory effects in benign prostate hyperplasia [27], and a protective effect in cerebral ischemia-reperfusion injury [28]. AEX is an acidic polysaccharide isolated from *Coccomyxa gloeobotrydiformi*. Monosaccharide composition analysis revealed that AEX contained galactose (Gal), mannose (Man), glucose (Glc), arabinose (Ara), xylose (Xyl) and rhamnose (Rha) [29]. Takayuki Komatsu et al. have demonstrated that AEX possesses antiviral activity against human influenza A virus infection in vitro [29]. However, its bioactivities, such as the immunomodulatory and antiviral activities of AEX in chicken, are still unknown.

This study analyzed the in vitro effects of AEX on cytokine production in chicken PBMCs and spleen lymphocytes, spleen lymphocyte proliferation, NO production and cytotoxicity. We also evaluated the in vitro and in vivo effects of AEX on IBDV replication in the chicken bursa of Fabricius and specific antibody levels in peripheral blood. 

## 2. Results

### 2.1. Effects of AEX on Cytokine and NO Production in Chicken PBMCs

**Figure 1:**
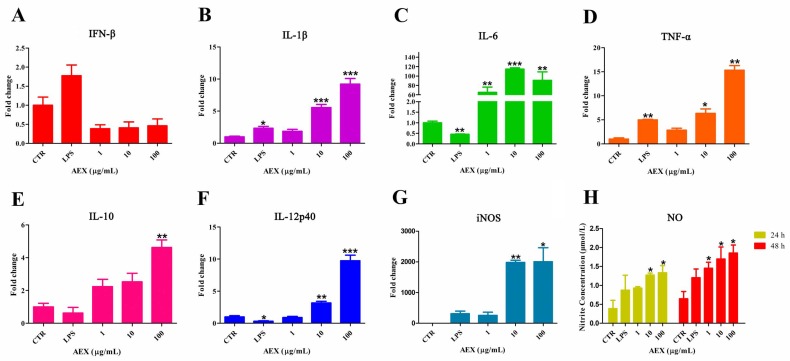
Multiple cytokines and iNOS expression were upregulated and NO production were increased by AEX in PBMCs. (**A**–**G**) PBMCs were isolated and cultured with AEX (0–100 μg/mL) or LPS (100 ng/mL) for 24 h. Then total RNA was extracted and analyzed by qRT-PCR for IFN-β, IL-1β, IL-6, TNF-α, IL-10, IL-12p40, and iNOS; (**H**) PBMCs were isolated and cultured with AEX (0–100 μg/mL) or LPS (100 ng/mL) for 24 h and 48 h. Then, the culture supernatants were collected and nitrite contents were determined by Griess reaction. Data represent means ± SEM from three wells per group. * *p* ≤ 0.05; ** *p* ≤ 0.01; *** *p* ≤ 0.001. Results are representative of two independent experiments.

Cytokines play a critical role in innate immunity and adaptive activation during infection. Inflammatory stimulation or cytokines can induce the expression of inducible nitric oxide synthase, which uses NADPH, O_2_, and Arg to synthesize NO. To assess the effect of AEX on cytokine and NO production in chicken PBMCs, the PBMCs were isolated from the peripheral blood of 4-week-old SPF chickens and then treated with AEX, LPS, or PBS at the indicated concentrations. Inflammatory cytokines (IFN-β, IL-1β, IL-6, TNF-α), T-helper cell differentiation cytokines (IL-10, IL-12p40), and iNOS mRNA levels were analyzed by qRT-PCR. Figure 1A–D shows that AEX significantly increased IL-1β, IL-6, and TNF-α mRNA levels in a dose-dependent manner but did not affect IFN-β, whereas LPS increased IFN-β mRNA expression by 1.8-fold. As shown in Figure 1E,F, IL-10 and IL-12p40 mRNA levels were also remarkably increased by AEX in a dose-dependent manner. In addition, the mRNA expression of iNOS was significantly increased by AEX (Figure 1G). Moreover, the NO concentration analysis showed that AEX also induced NO production in a dose-dependent manner (Figure 1H). These observations suggest that AEX can up-regulate various pro-inflammatory cytokines, T-helper cell differentiation cytokines, and NO production by activating chicken PBMCs.

### 2.2. Effect of AEX on Gene Expression in Splenic Lymphocytes

**Figure 2:**
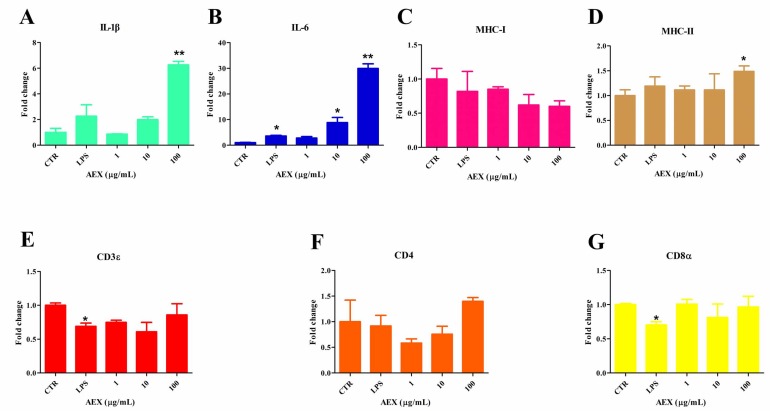
Inflammatory cytokines and surface molecules on splenic lymphocytes were regulated by AEX. Splenic lymphocytes were isolated and cultured with AEX (0–100 μg/mL) or LPS (100 ng/mL) for 24 h. Then, total RNA was extracted and analyzed by qRT-PCR for IL-1β (**A**), IL-6 (**B**), MHC I (**C**), MHC II (**D**), CD3ε (**E**), CD4 (**F**), and CD8α (**G**). Data represent means ± SEM from three wells per group. * *p* ≤ 0.05; ** *p* ≤ 0.01. Results are representative of two independent experiments.

As the major lymphoid organ for antigen presentation, the spleen contains a mass of DCs and T and B lymphoid cells. To investigate the effect of AEX on inflammatory cytokines (IL-1β and IL-6), major histocompatibility complexes (MHC I and MHC II), and T lymphocyte surface markers (CD3, CD4, CD8) mRNA expression in chicken splenic lymphocytes, the lymphoid cells were isolated from the chicken spleen and treated with AEX, LPS, or PBS at the indicated concentrations. As shown in Figure 2A,B, AEX significantly increased IL-1β and IL-6 mRNA expression in a dose-dependent fashion. MHC I mRNA levels were reduced by AEX treatment, whereas the expression of MHC II was not (Figure 2C,D), implying that AEX may negatively regulate the antigen presentation of viral infection between the antigen presenting cells (APCs) and the CD8^+^ T lymphoid cells. However, the expression of CD3, CD4, and CD8 mRNA was not affected by AEX treatment (Figure 2E–G). These data suggest that AEX can up-regulate inflammatory cytokines (IL-1β and IL-6) mRNA levels but down-regulate MHC I expression.

### 2.3. Effect of AEX on Lymphocyte Proliferation

**Figure 3:**
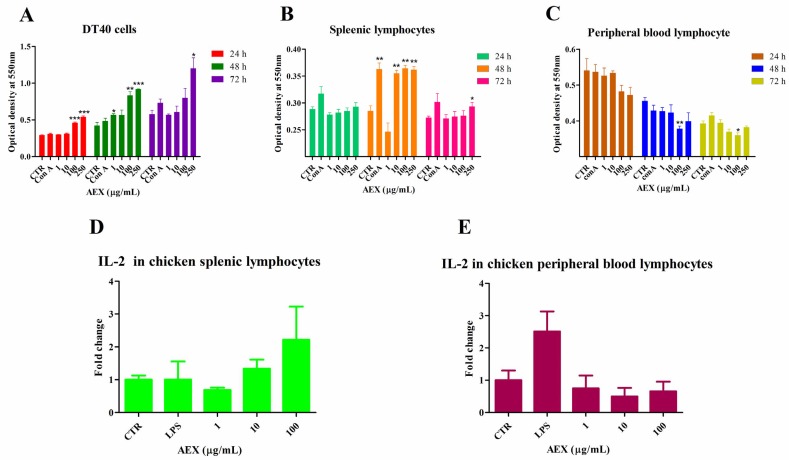
AEX promoted DT40 and splenic lymphocytes proliferation, but reduced peripheral blood lymphocyte proliferation in vitro. (**A**–**C**) DT40 cells, splenic lymphocytes and peripheral blood lymphocytes were cultured in 96-well plates. After being stimulated by AEX (0–250 μg/mL) or Con A (40 μg/mL) for 24 h, 48 h, and 72 h, respectively, the proliferation was examined by MTT method as described in the Materials and Methods section; (**D**,**E**) Splenic lymphocytes and peripheral blood lymphocytes were isolated and cultured with AEX (0–100 μg/mL) or LPS (100 ng/mL) for 24 h, respectively. Then, total RNA was extracted and analyzed by qRT-PCR for IL-2. Data represent means ± SEM from three wells per group. * *p* ≤ 0.05; ** *p* ≤ 0.01; *** *p* ≤ 0.001. Results are representative of two independent experiments.

To assess the effect of AEX on lymphocyte proliferation, we isolated splenic lymphocytes from 4-week-old SPF chickens and co-cultured them with AEX, Con A or PBS. The MTT assays indicate that AEX only slightly promoted splenic lymphocyte proliferation at 24 h and 72 h, but a significant promotion of proliferation was observed at 48 h in a dose-dependent manner (Figure 3B). The lymphocyte proliferation assay was also performed on the DT40 cells (chicken bursa of Fabricius derived cell line) and the peripheral blood lymphocytes. AEX significantly promoted the proliferation of the DT40 cells (Figure 3A). However, the proliferation of peripheral blood lymphocytes was reduced by AEX treatment in a dose-dependent manner (Figure 3C). Consistent with the lymphocyte proliferation effect, AEX up-regulated IL-2 mRNA levels in chicken splenic lymphocytes but down-regulated it in chicken peripheral blood lymphocytes (Figure 3D,E), suggesting that AEX may promote splenic lymphocyte proliferation by up-regulating IL-2 expression and inhibit peripheral blood lymphocyte proliferation by down-regulating IL-2 expression.

### 2.4. In Vitro Anti-Viral Effect of AEX on the IBDV TS Strain Virus

**Figure 4:**
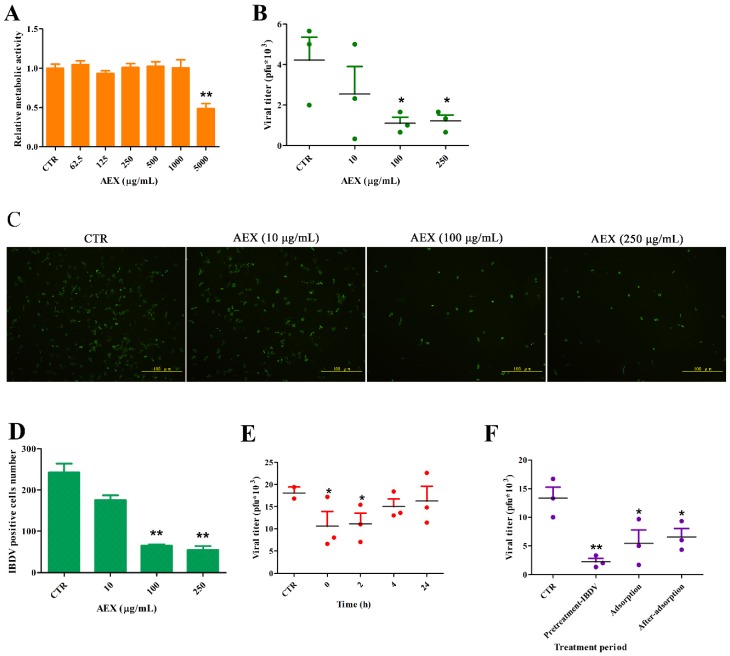
AEX inhibited IBDV replication by deactivating viral particle and interfering with adsorption. (**A**) The Vero cells were treated with different concentrations of AEX for 24 h and cell viability was measured by MTT assay; (**B**) The Vero cells infected with IBDV (MOI = 1.0) were treated with different concentrations of AEX, and viral titers in the supernatant were identified by TCID_50_ assays on the Vero cells. PBS treatment wells served as the control; (**C**,**D**) The Vero cells infected with IBDV (MOI = 1.0) were treated with different concentrations of AEX, and immunofluorescence staining was performed using anti-IBDV antibodies. (**C**) Scale bar represents 100 μm; (**D**) IBDV positive cells number was calculated; (**E**) The Vero cells were incubated with IBDV (MOI = 1.0) for 1 h and treated with AEX at the indicated time points (0, 2, 4, 24 h p.i.). Viral titers in the supernatant were determined by TCID_50_ assays on the Vero cells at 48 h p.i. (**F**) The Vero cells were infected with IBDV (MOI = 1.0) via different polysaccharide treatment method (IBDV pretreatment, adsorption, and after adsorption) and viral titers in the supernatant were determined by TCID_50_ assays on the Vero cells at 48 h p.i. Data represent means ± SEM from three wells per group. * *p* ≤ 0.05; ** *p* ≤ 0.01. Results are representative of two independent experiments.

First, we investigated the cytotoxicity of AEX on the metabolism of the Vero cells. The MTT assay showed that there was no observable difference in the metabolic activity of the Vero cells treated with 62.5–1000 μg/mL of AEX and the untreated cells. However, at a concentration of 5000 μg/mL, the AEX treatment reduced the relative metabolic activity by 62% (Figure 4A).

Then, we tested the in vitro anti-IBDV activity of AEX on the Vero cells. The Vero cells were first infected with the IBDV TS strain (MOI = 1.0) and then treated with compounds at the indicated concentrations after removal of the viral inoculum. After 48 h, the virus titers and the IBDV positive cells were identified by PFU and IFA assays. As shown in Figure 4B–D, AEX significantly reduced viral titers when used at a concentration greater than 100 μg/mL (*p* < 0.05). AEX reduced the IBDV positive cell number from 250 (CTR) to 50 at a concentration of 250 μg/mL. Furthermore, the influence of the treatment duration with AEX on IBDV multiplication was examined.

AEX was added to the Vero cells at different times after IBDV adsorption. As shown in Figure 4E, the viral titers were decreased by AEX only when it was added to the Vero cells at 0 and 2 h, whereas AEX showed no anti-IBDV effect when it was added at 4 and 24 h. Moreover, when IBDV was pretreated with AEX, the inhibitory effect was more than 70%. However, there was less of an inhibitory effect when AEX was added during adsorption (50%) or after-adsorption (50%) (Figure 4F). These data indicate that the antiviral activity of AEX is largely related to its deactivation of viral particles and inhibition of virus life-cycle early events that occur 0–2 h after adsorption.

### 2.5. In Vivo Anti-Viral Effect of AEX on the IBDV TS Strain Virus

**Figure 5:**
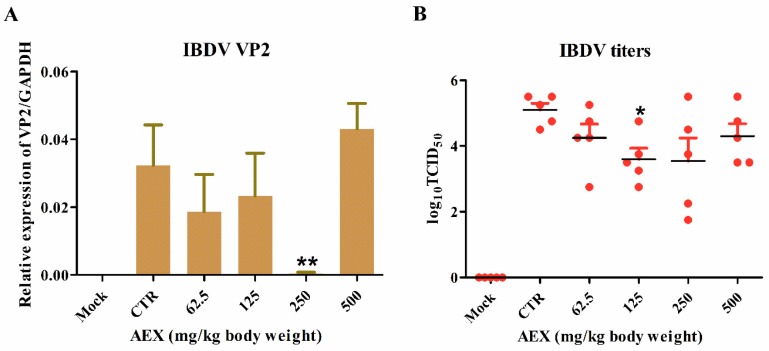
AEX inhibited IBDV replication in chicken bursa of Fabricius. SPF chickens (6 chickens per group) were oral inoculated with different concentrations of AEX (0, 62.5, 125, 250 and 500 mg/kg body weight) per day. After three days, chickens were eye-dropped with IBDV (10^5.5^TCID_50_). Bursa of Fabricius were collected at 5 d.p.i. and divided into two parts. (**A**) Half of the bursa of Fabricius were grinded and virus titers were determined by TCID_50_; (**B**) The other half bursa of Fabricius were subjected to isolate total RNA and the expression lever of IBDV *VP2* was evaluated by qRT-PCR. Data represent means ± SEM from five wells per group. * *p* ≤ 0.05; ** *p* ≤ 0.01. Results are representative of two independent experiments.

To further explore the therapeutic potential of AEX, SPF chickens were infected with 10^5.5^TCID_50_ of the IBDV TS strain and treated with AEX at the concentrations indicated in Figure 5. Oral administration of AEX was started 24 h post infection and repeated once daily for 3 days. As shown in Figure 5A,B, IBDV replicated efficiently in chicken bursa of Fabricius in the CTR group. AEX slightly decreased *VP2* mRNA expression at concentrations of 62.5 and 125 mg/kg body weight. When 250 mg/kg body weight of AEX was used, *VP2* mRNA was significantly reduced (Figure 5A). The viral titer in the bursa of Fabricius detection also showed that AEX decreased the IBDV viral titer at concentrations of 62.5 to 500 mg/kg body weight. (Figure 5B). 125 mg/kg body weight of AEX decreased the viral titer from 10^5.5^TCID_50_ (CTR) to 10^3.5^TCID_50_. These data suggest that AEX can suppress the replication of the IBDV TS strain in chicken bursa of Fabricius.

### 2.6. Effect of AEX on Anti-IBDV Antibodies in Immunized Chickens

**Figure 6:**
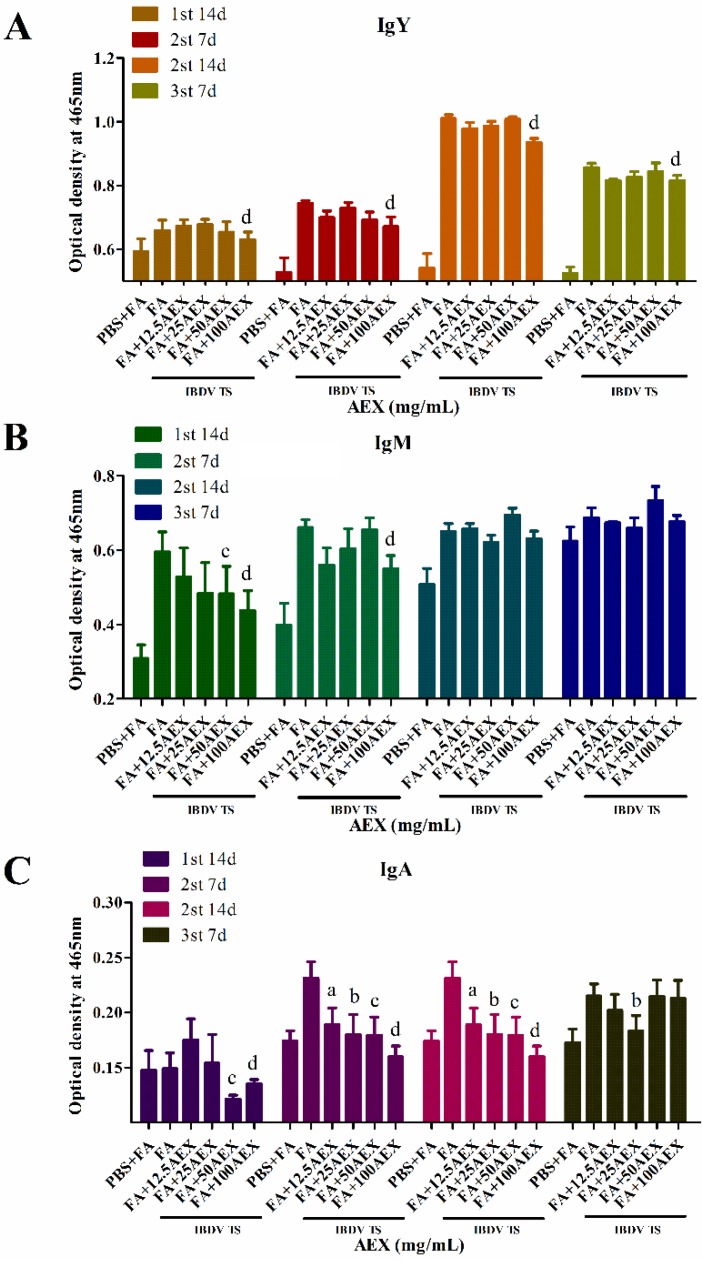
AEX reduced specific anti-IBDV antibody (IgY, IgM, and IgA) titers in the sera of chickens immunized with IBDV. SPF chickens (6 chickens per group) were immunized with IBDV vaccine by subcutaneous injection in the absence or presence of AEX (PBS used as control) thrice at a 14-day interval. The sera were collected (14 days after the first immunization, 7 days and 14 days after secondary immunization, and 7 days after tertiary immunization) and specific antibody (**A**) IgY; (**B**) IgM; (**C**) IgA were detected by ELISA. Data represent means ± SEM from five wells per group. ^a,b,c,d^ *p* ≤ 0.05 vs. *FA*. Results are representative of two independent experiments.

The B cells in chicken bursa of Fabricius are the major target of IBDV, which causes B cell apoptosis and depletion in the bursa of Fabricius to repress humoral immunity. To investigate the regulation of AEX on specific antibody titers against IBDV, we immunized SPF chickens with an intermediate IBDV vaccine every two weeks for a total of three times and detected the IBDV antibody titers (IgY, IgM, IgA) in the sera with an indirect ELISA. As shown in Figure 6, both the IgY and IgM antibody titers significantly increased after the first and second immunization and peaked 14 days after the secondary immunization. However, IgA antibody titers were only slightly increased after 7 days after the secondary immunization. Surprisingly, the IgY and IgM antibody titers in the IBDV immunized chickens were slightly decreased by AEX at concentrations from 12.5 to 50 mg/mL, and the IgY and IgM antibody titers of the FA + 100AEX groups were significantly lower than those of the FA + IBDV group (Figure 6A,B). In addition, AEX showed an inhibitory effect on the IgA antibody titers in a dose-dependent manner (Figure 6C). These results suggest that in some case AEX may negatively regulate specific antibody production when we immunized the chickens with live attenuated vaccines.

## 3. Discussion

AEX was previously reported to exert an antiviral effect on the influenza virus [29]. In this study, we investigated the immunoregulatory effect of AEX on chicken immune cells and the antiviral effect of AEX against IBDV, as well as the adjuvant effect of AEX. 

Immunomodulators are natural or synthetic substances that regulate or modify the function of the immune system [30]. According to their effects, Immunomodulators can be divided into immunostimulants and immunosuppressants. It has been reported that many polysaccharides have immunoregulatory activities [31]. Here, we observed that AEX significantly increased IL-1β, IL-6, TNF-α, and iNOS mRNA levels, and promoted the secretion of NO in chicken PBMCs. The activation of chicken PBMCs was characterized by the release of inflammatory cytokines and NO production [32,33,34], which implied that AEX could effectively activate chicken PBMCs. So AEX is an immunostimulant.

Interestingly, though, the mRNA expression levels of IFN-β were not regulated by AEX. As IRF3 is a transcription factor essential for IFN-β [35], we theorize that it may not be involved in the AEX receptor signaling pathway.

The activation of T cells generally requires a signal delivered by the interaction of the TCR with a specific antigen on the MHC molecules (MHC I and MHC II). By interacting with CD8 molecules on surfaces of cytotoxic T cells, MHC I mediates the destruction of intracellular antigens, especially virus infections, and establishes cellular immunity. MHC II mediates humoral immunity by interacting with CD4 molecules on the surface of T-helper cells. In this study, the expression of MHC I mRNA was down-regulated by AEX treatment whereas MHC II mRNA levels were increased, which implies that AEX promotes extracellular antigen presentation but negatively regulates intracellular antigen presentation. 

Lymphocytes proliferate extensively before they differentiate into functional effector cells of a particular specificity. Tang et al. demonstrated that Sophy β-glucans significantly improved the lymphocyte proliferative response of duck PBMCs and potentially enhanced the cellular immune response [36]. In the current study, the proliferation of both chicken primary splenic lymphocytes and DT40 cells, which are a chicken bursa of Fabricius lymphoma cell line, was significantly improved by AEX treatment. However, peripheral blood lymphocyte proliferation was decreased by AEX treatment. Considering that DT40 is a B lymphocyte line and splenic lymphocytes consists of both B and T lymphocytes [37], whereas most peripheral blood lymphocytes are T lymphocytes [38], AEX could improve the B lymphocyte proliferation but suppress the proliferation of T lymphocytes.

Astragalus polysaccharides, fucoidan, rape pollen polysaccharide, epimedium polysaccharide, and echinacea polysaccharide have been reported to show anti-IBDV activity and improve chicken immune response [39,40,41]. Here, we observed that AEX showed an in vitro anti-IBDV effect, especially when added at 0–2 h post infection or when pretreated with IBDV particles, which suggests that AEX can inhibit replication by inactivating IBDV particles. Our in vivo anti-IBDV assay also indicated that AEX can down-regulate IBDV *VP2* mRNA expression and viral titers when AEX was delivered by oral administration. Therefore, AEX could be a suitable alternative approach to therapy meant to address anti-IBDV.

Immunomodulators are potential agents for improving poultry immunity and growth performance [42]. Several glucans have been used as an adjuvant for vaccines [43,44,45]. Sulfated polysaccharides isolated from both *Grateloupia filicina*, *Ulva pertusa* and *Sargassum qingdaoense*, could increase H9N2-specific antibody titers in the sera of mice immunized with inactivated avian influenza virus [46]. Despite the fact that chicken PBMCs activation and lymphocyte proliferation were improved by AEX treatment, specific antibodies against IBDV in the sera of immunized chicken were decreased instead of showing improvement. That may due to the direct virucidal action of AEX. The vaccine we used in this experiment is an intermediate vaccine which has the similar action as normal IBDV. The AEX, as an effective virucidal substance, may affect the immunogenicity of the vaccine particles or block its surface antigen, which results in lower specific antibodies titers. To avoid that negative effect, we could try to find a suitable dosage between the promotion of producing antibody and the inhibition of vaccine particles, or we could inject AEX adjuvant before vaccines, lowering the negative impact on vaccines activity. In addition, we suppose that AEX may be a good choice as adjuvant for an inactivated IBDV vaccines.

## 4. Materials and Methods

### 4.1. Animals, Cells, Compounds and Reagents

Ten-day- and 4-week-old specific pathogen-free (SPF) White Leghorn chickens were purchased from Meria (Meria, Beijing, China) and raised in a laboratory animal house. Peripheral blood lymphocytes, spleen lymphocytes and DT 40 cells were maintained in RPMI-1640 medium (HyClone, Logan, UT, USA) supplemented with 10% fetal bovine serum (FBS; HyClone, Logan, UT, USA) and 1% antibiotics (Sigma, St. Louis, MO, USA). The Vero cells were purchased from ATCC and maintained in DMEM medium (HyClone, Logan, UT, USA) supplemented with 10% FBS and 1% antibiotics. The acidic polysaccharide of the *Coccomyxa gloeobotrydiformis* Nikken strain (AEX) was supplied by the Nikken Sohonsha Corporation (Hashima, Gifu, Japan). LPS and Concanavalin A (Con A) were purchased from Sigma-Aldrich (St. Louis, MO, USA).

### 4.2. Cell Isolation

The peripheral blood collected from 4-week-old SPF chickens was diluted with Ca^2+^- and Mg^2+^-free Hank’s balanced salt solution (1:1, vol/vol), carefully layered onto the Histopaque-1077 (Sigma) in 15 mL conical centrifuge tubes, and centrifuged at 400× *g* for 30 min at room temperature. The lymphocyte layer was collected, washed 3 times in Hank’s solution, and spun at 250× *g* for 10 min. Then, the cells were resuspended in RPMI-1640 medium containing 10% FBS and cultured for 2 h. The suspension cells (peripheral blood lymphocytes) were collected and used for the lymphocyte proliferation assay, whereas the anchorage-dependent cells (peripheral blood molecular cells) were used for the cytokine and NO analyses. 

The spleens from the 4-week-old SPF chickens, sacrificed under aseptic conditions, washed with Hank’s solution and crushed to isolate the spleen cells. The spleen cell mass was passed through a 200 mesh copper sieve to obtain a homogeneous cell suspension. Spleen lymphocytes were isolated from the suspension using the same method as that used for the isolation of the peripheral blood lymphocytes.

### 4.3. Lymphocyte Proliferation Assay

Isolated peripheral blood lymphocytes, spleen lymphocytes and DT 40 cells were resuspended in RPMI-1640 medium containing 10% FBS and adjusted to 1 × 10^7^ cells/mL. The lymphocytes were distributed (100 μL per well) onto 96-well plates (Costar, Corning, Grand Island, NY, USA) and treated with different concentrations of AEX (1, 10, 100, and 250 μg/mL) for 24 h, 48 h and 72 h, respectively. Con A served as the positive control. Lymphocyte proliferation activity was tested by a 3-(4,5-dimethylthiazol-2-yl)-2,5-diphenyl tetrazolium bromide (MTT; Sigma, St. Louis, MO, USA) assay [47]. 10 μL of PBS containing MTT (final concentration: 0.5 mg/mL) was added to each well. After a 4 h incubation at 37°C, the absorbance values were measured in a microplate reader (Bio-Rad, Hercules, CA, USA) at 550 nm.

### 4.4. Nitric Oxide (NO) Production Assay

After a 24 h treatment with various concentrations of AEX, the culture supernatants of the PBMCs were collected, and the nitrite contents were determined by the Griess reaction using the Griess Reagent System (Promega, Madison, WI, USA) according to the manufacturer’s protocol.

### 4.5. qRT-PCR Analysis

Total RNA was first extracted from the AEX-treated PBMCs, spleen lymphocytes and bursa of Fabricius using a TRIgene (GeneStar, Beijing, China) and treated with Dnase I (Promega, Madison, WI, USA) to remove the DNA. Then, 1 μg of RNA was reverse-transcribed into cDNA using a GoScript reverse transcription system (Promega, Madison, WI, USA) in a 20 μL reaction mixture. Quantitative real-time PCR amplification was performed in 15 μL of the LightCycler^®^ 480 SYBR Green I Master Mix (Roche, Rotkreuz, Switzerland) with the LightCycler^®^ 480 Real-time PCR System (Roche, Rotkreuz, Switzerland). The individual primers used were designed by Primer Express 3.0 and are shown in Table 1. The qRT-PCR was performed under the following cycling conditions: 95 °C for 10 min, 40 cycles of 95 °C for 15 s and 60 °C for 1 min, and one cycle of 95 °C for 15 s, 60 °C for 15 s, 95 °C for 15 s and 40 °C for 30 s. The relative mRNA abundances were calculated using the 2^−∆∆Ct^ method with GAPDH as a reference and plotted as the fold changed relative to the control samples.

### 4.6. Viruses and Infection

The IBDV Ts strain was amplified in the Vero cells, and the viral titers were determined as TCID_50_ by IFA on the Vero cells as previously described [50]. For the virus infection, the virus propagation solution was diluted in DMEM and was added to the cells at the indicated multiplicity of infection (MOI). The virus was allowed to adsorb for 1 h at 37 °C. After removing the virus inoculum, the cells were maintained in infective media (DMEM) at 37 °C in 5% CO_2_. All of the experiments with the IBDV Ts strain were performed under biosafety level 2 (BSL-2) conditions with investigators wearing the appropriate protective equipment and complying with the general biosafety standards for microbiological and biomedical laboratories of the Ministry of Health of the People’s Republic of China (WS 233-2002).

### 4.7. Cytotoxicity Assay

Cell viability was measured by an MTT assay. The Vero cells were seeded into 96-well plates (1 × 10^5^ cells/well) overnight. Subsequently, the medium was supplied with different concentrations of AEX or LPS. After a 24 h incubation at 37 °C and 5% CO_2_, 10 μL of PBS containing MTT (at a final concentration of 0.5 mg/mL) was added to each well for 4 h. After 4 h of incubation at 37 °C, the supernatant was removed, and 200 μL of DMSO was added to each well to solubilize the formazan crystals. After a vigorous shaking, the absorbance values were measured in a microplate reader at 570 nm.

### 4.8. Antiviral Assay (TCID_50_)

Antiviral activity was evaluated by the TCID_50_ assay in Vero cells. The Vero cells were incubated with the IBDV at an MOI of 1 for 1 h at 37 °C and washed to remove the unbound virus; then, the infecting media containing different concentrations of AEX was added to the cells, which were incubated at 37 °C for 48 h. Subsequently, the supernatant was removed and collected, 10-fold serially diluted, and incubated with the Vero cells for 36 h. The TCID_50_ was determined according to Reed and Muench (1938) [51]. 

### 4.9. Immunization of SPF Chicken

Five-day-old SPF chickens were randomly divided into 6 groups (*n* = 6/group) and immunized with different concentrations of AEX and IBDV live vaccine (strain B87, Lufang Biology, Yangling, Shaanxi, China). Immunization of chickens with IBDV and AEX was performed as described previously [52]. For the first injection, vaccine (following the dosing recommendations in the labeling) and different doses of AEX (0, 12.5, 25, 50, 100 mg/mL) were solved in PBS and emulsified with an equal volume of complete Freund’s adjuvant (FA, Sigma). Each chicken received 0.1 mL emulsion by subcutaneous injection in the neck. Booster injections were given subcutaneously in the neck region 2 week and 4 week after the first injection respectively with the same dose emulsified with incomplete FA (Sigma).

### 4.10. Measurement of Specific Antibody in the Sera of Immunized Chickens

The sera samples were collected from immunized chickens 14 days after the first immunization, 7 days and 14 days after the secondary immunization, and 7 days after the tertiary immunization. Then, specific antibodies were detected by ELISA. Briefly, flat-bottom 96-well ELISA plates (Costar, Coring, NY, USA) were coated with IBDV (TCID_50_ of 10^5.5^/0.1 mL) in 0.05 mol/L carbonate-bicarbonate buffer pH 9.6 for 24 h at 4 °C. The wells were washed three times with phosphate-buffered saline (PBS) containing 0.05% (*v*/*v*) Tween 20 (PBST), and blocked with PBS containing 1% gelatin at 37 °C for 1 h. After washing the wells with PBST three more times, 100 μL of a series of diluted sera from immunized chickens or PBS containing 0.1% gelatin were added to the triplicate wells as control. The plates were then incubated for 1 h at 37 °C, followed by being washed with PBST, 100 μL of goat anti-chicken IgG-HRP, IgM-HRP, and IgA-HRP (Cwbio, Beijing, China) (diluted 1:5000 with PBS containing 0.1% gelatin, respectively) were added to each plate. The plates were further incubated for 1 h at 37 °C. Substrate 3,3′,5,5′-tetramethylbenzidine (TMB) was added to each well after being washed with PBST, and the plate was incubated for 10 min at room temperature. Reaction was terminated by adding 50 μL of 2 mol/L H_2_SO_4_ to each well, and optical density (OD) was detected at 465 nm with a microplate reader (Bio-Rad, Hercules, CA, USA). 

### 4.11. Statistics

All data analyses were performed using SPSS 16.0 (SPSS Inc., Chicago, IL, USA). One-way ANOVA was used to detect significant differences between compound-treated groups and control groups. *p* values < 0.05 were considered significant. Data are presented as mean ± SEM from at least three wells per group. Results are representative of two independent experiments.

### 4.12. Ethics Statements

This study was approved by the Laboratory Animal Care and Use Committee of the China Agricultural University (Permit Number: SKLAB-2014-06-06). All efforts were made to minimize animal suffering and the number of animals used. 

## 5. Conclusions

In summary, our study first reported that AEX, derived from *Coccomyxa gloeobotrydiformis*, activates chicken PBMCs by up-regulating cytokine and NO production, promotes chicken splenic lymphocyte proliferation, and inhibits IBDV replication by interfering with the early events of IBDV replication or inactivating IBDV particles in vitro and in vivo. AEX is a promising antiviral candidate for the prevention or treatment of IBDV in the poultry industry. However, it has some limitations and still needs more testing to find suitable conditions for use as an adjuvant for the IBDV vaccine.

## Figures and Tables

**Table 1:** Sequence of the primers used in qRT-PCR.

Genes	Direction	Sequence	Accession NO. in GenBank
GAPDH ^a^	Forward	TGCCATCACAGCCACACAGAAG	AF047874.1
	Reverse	ACTTTCCCCACAGCCTTAGCAG	
IFN-β	Forward	ACAACTTCCTACAGCACAACAACTA	X92479.1
	Reverse	GCCTGGAGGCGGACATG	
IL-1β	Forward	TTGCTGGTTTCCATCTCGTATGTA	NM_204524
	Reverse	CCCAGAGCGGCTATTCCA	
IL-2 ^b^	Forward	TTCTGGGACCACTGTATGCTCTT	AF000631.1
	Reverse	TACCGACAAAGTGAGAATCAATCAG	
IL-4 ^b^	Forward	AATGACATCCAGGGAGAGGTTTC	AJ621249.1
	Reverse	AGGCTTTGCATAAGAGCTCAGTTT	
IL-6 ^a^	Forward	GACGAGGAGAAATGCCTGACG	AJ309540.1
	Reverse	CCGAGTCTGGGATGACCACTTC	
IL-10 ^b^	Forward	GCTGAGGGTGAAGTTTGAGGAA	AF000631.1
	Reverse	GAAGCGCAGCATCTCTGACA	
IL-12p40 ^b^	Forward	CGAAGTGAAGGAGTTCCCAGAT	AY262752.1
	Reverse	GACCGTATCATTTGCCCATTG	
TNF-α	Forward	GGAATGAACCCTCCGCAGTA	AY765397.1
	Reverse	CACTACGGGTTGCTGCACAT	
iNOS	Forward	GCCCCTCCAGCTGATCAGA	D85422.1
	Reverse	AGGCCTGTGAGAGTGTGCAA	
CD3ε	Forward	TGCCAAAGTGTGTGCAAACTG	NM_206904.1
	Reverse	AGTACCCCCAAGGTGATGAGAA	
CD4	Forward	GCTGTGTGTTTGCGGTCATC	Y12012.1
	Reverse	CCTTTCCTGCAATCCCAATC	
CD8α	Forward	CGACAATGGTGTCTCCTGGAT	NM_205235.1
	Reverse	GGGAAAGTGGTCCGGGATAA	
MHC-I	Forward	TGGTTGGTGTTGGATTCATCAT	KF294514.1
	Reverse	GCTGGATCCACCTTCCTTGTC	
MHC-II	Forward	GTGGGCTCAGTTCGGTTTTC	DQ207939.1
	Reverse	AATTCGGGCAGCCTCCATA	
TS vp2 ^b^	Forward	ACCGGCACCGACAACCTTA	AF076230.1
	Reverse	CCCTGCCTGACCACCACTT	

^a^ Primers from Reference [48]; ^b^ Primers from Reference [49].
